# An updated synthesis of environmental factors influencing mobility and social participation in older adults: results from a scoping review

**DOI:** 10.1186/s12889-025-25849-5

**Published:** 2025-12-04

**Authors:** Laurie Paulhus, Kaylee Bernard, Charlotte Bourque, Simon Guilmette, Marie-Êve Laquerre, et Laurie-Ève Laramée, Mélanie Levasseur

**Affiliations:** 1 School of Rehabilitation, Faculty of Medicine and Health Sciences, Program of Occupational Therapy, University of Sherbrooke, 3001 12th Avenue North, Qc J1H 5H3 Sherbrooke, Canada; 2 Research Centre on Aging, Health, and Social Services Centre, University Institute of Geriatric of Sherbrooke (CSSS-IUGS), Sherbrooke, Québec Canada

**Keywords:** Aged, Aging in place, Community resources, Independent living, Locomotion, Neighborhood characteristics, Movement, Scoping review, Social cohesion, Social environment

## Abstract

**Background:**

As population ages globally, understanding how to better support mobility and social participation is increasingly important. Although several studies have explored the associations between the neighborhood environment, mobility and social participation, no integrated synthesis of current evidence is available yet. This study aimed to understand how the neighborhood environment currently affects mobility and social participation in older adults.

**Method:**

A scoping review followed a rigorous methodology based on the PRISMA guidelines. Seven databases were searched using a predefined set of keywords to retrieve empirical studies published between October 2013 and February 2025. Relevant articles were retained based on clear inclusion (inform about how the neighborhood environment influences mobility and social participation of individual aged 60 and older in high income country) and exclusion criteria (too narrow or specific, not from empirical study). Data was extracted, categorized and synthesized in Tables 2 and 3.

**Results:**

Of the 38 selected articles, the majority report results of qualitative studies (16/38; 42.1%), quantitative (12; 31.6%) and others were mixed (10; 26.3%). Mainly conducted in Canada (7; 18.4%), most of these studies examined how the neighborhood environment is associated with both mobility and social participation (49; 63.63%). Among the 77 neighborhood attributes considered, they mainly focused on ‘Products and technology’ (32/77; 41.6%), ‘Services, systems and policies’ (17; 22.1%) and ‘Natural and human-made changes’ (17; 22.1%). These attributes mainly concerned ‘Access to amenities and services’, ‘Adequate public transportation’, ‘Nature and green space’, ‘Social cohesion’, ‘Gathering places’, ‘Aesthetics’, ‘Social support’ and ‘Neighborhood security’.

**Conclusions:**

Mobility and social participation of older adults are highly related and often influenced by the same attributes. Results suggest potential interventions, such as creating supportive environments that enable people to continue doing what matters to them. Despite a rigorous process, relevant studies might have been overlooked, and time constraint limited both the scope of this study and inclusion of knowledge-users. Further research should consider a wider range of settings and focus on underexplored attributes to better promote mobility and social participation among older adults.

## Background

The proportion of the population aged 65 and older is growing worldwide, and, according to the World Health Organization (WHO), will nearly double by 2050 [1]. As demographic changes, health conditions common among older adults, such as hearing loss, cataracts, and other vision impairments, back and neck pain, osteoarthritis, chronic obstructive pulmonary disease (COPD), diabetes, depression, and dementia, are becoming increasingly prevalent [1]. Chronic illness can affect functional independence and have other detrimental consequences including dying prematurely [2]. As adopting healthy lifestyle behaviors, such as exercising regularly, is associated with a lower risk of chronic illnesses [2], it becomes essential to have a better understanding of the factors that enable older adults to maintain an active and socially engaged life in their neighborhood and community. In addition to promoting active ageing, i.e., the process of optimizing opportunities for health, participation, and security to enhance quality of life as people age [3], such understanding is notably based on research focusing on the influence of the environment on mobility and social participation.

This understanding requires examining not only individual capacities but also the influence of the environment on mobility and social participation. Mobility is essential as it allows older adults to maintain their independence, participate in activities, engage in social and community life, and access resources [4]. In adults and older adults, regular mobility is associated with a reduced risk of all-cause mortality, cardiovascular disease mortality, incident of hypertension, site-specific cancers, type 2 diabetes, and falls, as well as improved mental and cognitive health [5]. Mobility encompasses more than just functional or utilitarian travel, it also includes movements driven by pleasure, independence, status, or identity [6]. For older adults, mobility plays a crucial role in well-being, as it is closely linked to a sense of control and overall quality of life [6]. Restricted mobility can lead to social isolation, depressive episodes [6], and a loss of functional independence [6, 7]. To continue participating according to their preferences, older adults can adopt strategies to overcome environmental barriers limiting their mobility, but these obstacles sometimes lead them to modify or even abandon certain activities [8]. Environmental factors, such as the accessibility of infrastructure and public transportation, quality of sidewalks, and neighborhood safety, play a key role in mobility among older adults, which in turn influences social participation [9, 10]. As activities such as walking in the neighborhood or attending community events can foster spontaneous social interactions, while maintaining social relationships can encourage greater mobility and reduce sedentary time [11], these concepts are closely linked.

Another important outcome is social participation, which can be defined as ‘*a person’s involvement in activities providing interactions with others*’ [12] ‘*in community life and in important shared spaces*,* evolving according to available time and resources*,* and based on the societal context and what individuals want and is meaningful to them.*’ [13]. Promoted by the World Health Organization as a key recommendation in active aging [14], social participation is associated with quality of life [11, 15] and health [12, 16] in older adults. Social participation and connection have also been found to have a protective effect on various health outcomes such as cancer [17], cognitive decline [18, 19], depression [20–23], anxiety, Alzheimer [24], obesity [25], and heart disease [26]. Furthermore, older adults having stronger social ties have a 50% increased likelihood of survival compared to those who are isolated [27]. Even if highly valued by some older adults and providing a sense of fulfillment [25], social participation might decline with age [16], notably due to loss of functional independence [28] or environmental barriers [29]. Given the impact of social participation on health [30], it is essential to create environment that better support older adults’ mobility and social participation.

As defined by the International Classification of Functioning, Disability and Health (ICF), the environment refers to the physical, social, and attitudinal characteristics in which individuals live and evolve. The ICF divided environment into five domains (chapters), with the first 2 chapters referring to physical features, the last 3 to social features: (1) ‘Products and technology’, (2) ‘Natural environment and human-made changes’, (3) ‘Support and relationships’, (4) ‘Attitudes’, and (5) ‘Services, systems and policies’ [31]. This conceptualization influences the neighborhood environment, which refers to the community where someone lives, including its physical, environmental, and societal conditions, and its impact on individual and family health and well-being [32]. A favorable neighborhood, accessible and with safe transportation and travel options, can allow older adults to maintain functional independence and participating in meaningful activities, thereby achieving good social participation [1, 33]. Published in 2015, one scoping review of 50 studies highlighted the importance of proximity to resources, social support, transportation and neighborhood security for mobility and social participation in older adults [11], two crucial outcomes for active aging.

Although several articles focused on how environment influences mobility and social participation, no current integrated knowledge is available yet. Considering global events and associated social and environmental changes in the last decade, such as the COVID-19 pandemic, digital era and climate changes, a recent integration is highly needed. Characterized by the use of masks, mandatory curfews, physical distancing, and closure of public places to reduce the spread of the virus, these imposed protective measures also contributed to isolation, simultaneously reducing the mobility [34] and social participation [35] of older adults. The recent acceleration of technological development has introduced new modes of interaction and social participation, such as telecommuting, online public services, and digital social networks [36]. While these advances have facilitated the maintenance of social contact, they have also excluded certain groups, particularly those unfamiliar with digital technologies [37]. Extreme heat waves, fires, floods, and other manifestations of climate change have intensified [38] and are impacting the entire population, especially vulnerable populations, including older adults, for whom health, safety and mobility may be compromised [39]. Following storms or other serious weather events, the relocation of buildings and creation of new installations has been mandatory, which can lead to neighborhood transformations and losses of sense of belonging [40]. Certain populations have also been forced to migrate to new unknown countries with safer climate, which can have a major influence on mobility and social participation, such as breaking of existing social ties [41]. Indeed, social isolation is linked to a deprived social participation, which can also restrict a person’s mobility [42]. Although fewer health promoting studies focused on the occupations developing older adults’ capacity compared to other population [43], one study found that plasma proteins were associated with diseases, especially inflammatory and cardiovascular problems, supporting that social connections could have both tangible biological and public health benefits [44]. To support decision-making and develop innovative interventions within that context, clear guidelines, and good practices for creating convivial neighborhood environments that promote mobility and social participation among older adults [11], an update integrated synthesis is highly needed. This study thus aimed to answer the following research question: ‘*What is the influence of the neighborhood environment on the mobility and social participation of older adults?*’.

## Methods

### Study design and data collection of relevant studies

To answer the research questions, a scoping study [45, 46] was conducted to update and synthesize evidence that has emerged since the publication of the previous review [11]. This scoping study aimed to identify gaps in the existing literature and examine the extent, range, and nature of recent research activity on how neighborhood environments influence mobility and social participation in older adults. The authors retrieved empirical studies, both quantitative and qualitative, published between October 2013 and February 2025. Expanding on the previous review [11] with complex combination strategies (truncation and proximity operator) to broaden the results, the search strategy was based on a combination of 4 keyword blocks (*Neighborhood environment*,* mobility*,* social participation and older adults*) in the *title* and *abstract* fields (Table 1).

**Table 1:** Selected key words

Keywords: [strategy**: S1 AND S2 AND S3 AND S4)]	S1	((environment* OR surrounding* OR area* OR space*) N1 (buil* OR physical* OR design* OR healthy OR living OR urban* OR suburban* OR rural* OR social* OR local* OR residential*)) OR neighbourhood* OR neighborhood*
	S2	((transport* OR transit*) N1 (public* OR alternativ* OR inclusiv* OR activ* OR urban OR access* OR system*)) OR mobilit* OR walk*
	S3	((communit* OR social* OR group* OR civic*) N1 (participat* OR engag* OR involv* OR isolat* OR integr* OR contact* OR activit* OR inclusion* OR inclusiv* OR connection* OR interaction* OR exclusion* OR collaborat* OR cooperat*))
	S4	(old* N1 (adult* OR people OR person* OR patient* OR communit*)) OR elder* OR senior* OR geriatric* OR gerontolog* OR ageing OR aging OR aged

**Fig. 1:**
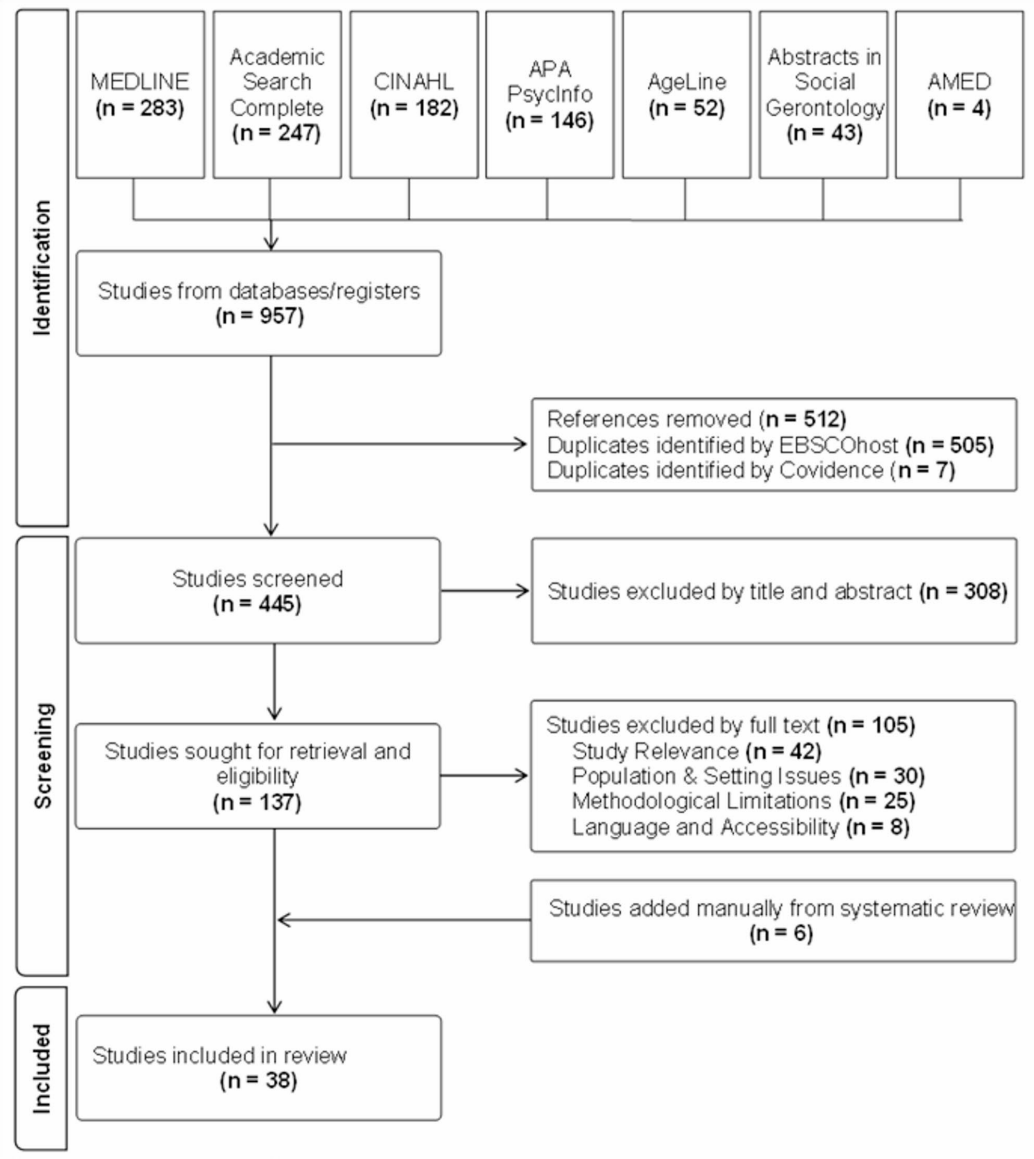
Flow chart

Based on recommendations from a university librarian, EBSCOhost was used to search in the following 7 main public health’s databases: (1) CINAHL Plus with Full Text, (2) Abstracts in Social Gerontology, (3) Academic Search Complete, (4) AgeLine, (5) Allied and Complementary Medicine Database (AMED), (6) APA PsycInfo and (7) MEDLINE with Full Text. The duplicates were eliminated automatically (Fig. 1).

### Inclusion and exclusion criteria of the selected studies

This study was conducted during an occupational therapy master’s degree program. While addressing a broad topic within a limited time, inclusion criteria were defined to restrict the search to the most relevant articles that: (1) explicitly mention the associations or influences of the neighborhood environment on mobility or social participation; (2) are in English or French, two languages in which the research team is proficient, to ensure rigorous data extraction; (3) are published between October 2013 and February 2025 to update a previous scoping review [11] that had been carried out with sources prior to this period; (4) studied a target population aged on average 60 or older, as used by the WHO [1]; and (5) come from a high income country, as classified by the World Bank Group [47] and cross-checked with the Human Development Report 2023–24 of the United Nations Development Programme [48]. Articles were excluded if: (1) the concepts or variables were too narrow or part of an immediate living environment (e.g., artificial intelligence, smart homes, situation of a single retirement home); (2) targeted a very specific population (e.g., chronic pain, dysphagia, visual problems, spinal cord injuries or severe cognitive impairment); (3) involved an exceptional context or socio-health emergency such as COVID-19; (4) did not provide results from an empirical study (expert opinions, conference proceedings). As they already provide synthesized results, scoping and systematic reviews were analyzed separately to manually identify other empirical studies.

### Data analysis: selecting the studies, charting the data, and collating, summarizing and reporting results

Covidence [49] was used to support double screening of first title and abstract, and next full text, i.e., random review association of two team members and the review was distributed equally among all 6 team members. To ensure transparency and reproducibility of the research process [45, 46], all the selected studies were identified on a flowchart following the PRISMA guidelines [50] considering its extension [51]. Weekly team meetings between the six masters’ students were held at every step of the process and the principal investigator met with the team on a monthly basis, allowing the team to discuss and clarify any ambiguities regarding the selection of studies, extraction and compilation of data, and synthesis and communication of the results. An inductive-deductive analysis approach was used with an evolving data charting form inspired from previous reviews [45, 46], but specifically for the present study, and the classification of the environmental factors from the ICF (Appendix 1) [31] and previous scoping review [11] were used to independently classify the extracted data. The influence of attributes of the environments had to be explicitly mentioned in the results of the selected articles to be categorized as influencing positively (+), negatively (-) or neutrally (0). The students followed a rigorous approach to analyze the data [45, 46] grouping categories by meaning, then synthesizing and organizing them into coherent, relevant, clearly defined, and informative themes. Some 2015 attributes [11] were reused, while others were revised or created to best fit the collected data, and reduce redundancy. To retain most relevant attributes, data associated with mobility and/or social participation from at least six articles were considered significant.

## Results

From the 957 articles retrieved through electronic search, 308 were excluded during a preliminary screening of the 445 articles initially selected by readings of abstracts and titles (Fig. 1). An in-depth reading of the remaining articles (*n* = 137) was then performed, 6 relevant empirical studies were manually added, leading to a final sample of 38 articles. Most of the articles reported results from qualitative studies (16/38; 42.1%), including one clinical randomized trial (1; 2.6%); quantitative studies (12; 31.6%), such as cross-sectional (7; 18.4%) and longitudinal designs (4; 10.5%); and a minority of mixed-methods studies (10; 26.3%). They were mainly conducted in Canada (7; 18.4%), the United Kingdom (6; 15.8%), the United States (5; 13.2%) and other countries (20; 52.6%; Table 2). The studies focused on the neighborhood environment and mobility (13; 34.2%), social participation (8; 21.1%); or both (17; 44.73%). Unlike the previous scoping published in 2015 [11] in which the majority of the results focus only on mobility, this review presents a better distribution with social participation and more studies addressing both concepts. Most studies (15; 39.5%) involved 150 participants or fewer. Studies with 150–500 participants (11; 29.0%) and those with 1,000 or more participants (11; 29.0%) each represented less than one-third of the total. The included studies’ settings were carried out in urban (21; 55.3%), mixed (13; 34.2%), suburban (2; 5.3%) and rural (2; 5.3%; Table 2) settings.

**Table 2:** Characteristics of the articles on neighborhood environment, mobility and social participation in older adults

Reference number	Country	Setting	Design	Population (sample size; age; median*; (mean age))	Objective
[9]	United States	Urban	Mixed	(638; 65 +)	To explore the intricate relationships between neighborhood walkability, third place engagement, and socio-demographic characteristics and their combined influence on the frequency of leisure-time physical activity and social capital among older adults.
[52]	Singapore	Urban	Qualitative	(25; 65+)	To use a participatory methodology to explore the determinants of an age-friendly neighborhood-built environment that promotes or limits healthy ageing in place among seniors residing in a low-income urban community in Singapore.
[53]	United States	Urban	Mixed	(35; 65+)	To evaluate the key elements to finding one’s way back in order to ensure the mobility and participation of seniors in urban areas.
[54]	Singapore	Urban	Qualitative	(12; 55–80)	To explore the neighborhood environmental factors that influence older adults’ out-of-home behaviors (OOHBs) in Yuhua East, Singapore.
[55]	Singapore	Urban	Qualitative	(30; 55 +) 63.3% were ≥ 65 years old	To examine how the physical and social neighborhood environment influences older adults’ physical activities, social participation, and their intersection (i.e., when both occur together).
[56]	Canada	Urban	Mixed	(15; 60 +)	To explore the factors that influence access to and use of urban greenways among older and disadvantaged adults in Québec City, Canada.
[57]	Canada	Urban + Rural + Suburban	Quantitative (Cross-sectional)	(4613; 65 +)	This study explored associations between neighborhood characteristics and frequency of participation in three social activities among older adults and interactions between neighborhood characteristics and mobility limitation as they relate to participation.
[58]	China	Urban	Qualitative	(38; 60 + (72.8))	To investigate older adults’ perceptions of their walking experiences using the social-ecological model as a framework.
[59]	Australia	Urban	Qualitative and clinical randomized trial	(315; 65 +)	To investigate relationships between health, fall-related risk factors, perceived neighborhood walkability, and walking behavior in older adults.
[60]	Canada	Urban	Qualitative	(6; 77–89; 82*; (82.5))	To understand how neighborhoods as physical and social environments influence community mobility.
[61]	Singapore	Urban	Mixed	(402; 55+; (69.1))	To assess the use of both perceived assessments and objective GIS measures of the neighborhood environment to examine their independent and combined associations with transportation physical activity among community dwelling elderly in Singapore.
[62]	Denmark	Urban	Mixed	(353; 50–90 (66.7))	To investigate the relationship between built environment features, social interaction, and walking activity among older adults in Neighborhood Open Spaces (NOS) in a low socio-economic neighborhood in Copenhagen.
[63]	Canada	Urban	Quantitative (Longitudinal)	(16 735; 65 +)	To explore the links between the residential neighborhood and the social participation of older people.
[64]	Canada	Urban + Rural + Suburban	Qualitative	(28; 65 +; (73.3))	To explore whether there is merit in the components and relationships described in the LEAAF among community-dwelling older adults.
[65]	United Kingdom	Urban + Rural	Qualitative	(14; 75–88)	To understand how older adults (over 70) interact with their local neighborhoods using a qualitative geographical information systems (QGIS) approach.
[66]	Israel	Urban + Rural	Mixed	(263; 65 +)	To examine the different variables that can have an impact on social participation, i.e. the environment and mobility, in addition to comparing rural and urban areas.
[67]	Portugal	Urban	Mixed	(850; 60 +; (71.7))	To explore the integration of sustainable mobility and universal design principles in the co-design of an accessible bus stop at Faro International Airport, with input from older adults and people with disabilities.
[68]	Australia	Suburban	Qualitative	(10; 64–83; 72*)	To identify enablers and barriers to participation in community-based activities experienced by active older adults.
[69]	New Zealand	Rural	Qualitative	(15; 85+; (88.1))	To understand the influence the physical and social environments have on enabling those aged 85 years and over to remain engaged in a rural community.
[70]	South Korea	Urban	Qualitative	(46; 65 +; (75.4))	To assess the attributes of a perceived urban neighborhood environment for the physical activity (PA) of older adults by applying a qualitative multimethod approach to collect both descriptive and spatial information.
[71]	China	Urban + Rural	Quantitative (Longitudinal)	(8408; 50 + (61.7))	To investigate the associations between residential greenness and the risk of disability in older adults in China.
[72]	Australia	Rural	Qualitative	(23; 50 + (76.2))	To explore the barriers and facilitators to social participation and the experience of loneliness among older adults in a rural Australian setting, from both individual and organizational perspectives.
[73]	Japan	Urban	Quantitative (Cross-sectional)	(214; 59–94; (73.8))	To investigate the association of built environments with frequency of going outdoors among older community-dwelling adults in Japan.
[74]	Belgium	Urban + Rural + Suburban	Mixed	(50 986; 65 +; (74.3))	To investigate the relationship between the perceived social environment and daily walking for transportation in older adults, while adjusting for individual and perceived physical environmental factors.
[75]	Germany	Suburban	Qualitative	(2559; 60 +)	To examine the association between frailty and individual, physical, and social environmental factors among Chinese older adults.
[76]	Canada	Urban	Quantitative (Cross-sectional)	(213; 65 +)	To examine the association between neighborhood-built environment (specifically, walkability) and physical activity habits especially walking for transportation among older adults of low socioeconomic status.
[77]	Netherlands	Urban + Rural	Quantitative (Cross-sectional)	(213; 65 +)	To analyze the heterogeneity in older adults’ preferences for different types of social activity locations (e.g., at home, community centres, or public ‘third places’) and to investigate how these preferences relate to personal and mobility characteristics.
[78]	United Kingdom	Urban + Rural	Quantitative (Longitudinal)	(6450; 65 +)	To investigate the factors associated with weekly walking hours among older adults in the UK using the socioecological model of health.
[79]	United Kingdom	Urban + Rural	Quantitative (Longitudinal)	(371 220; 65 +)	To make great connections between the different types of neighborhoods and the use of public transport, therefore the mobility of seniors.
[80]	United States	Urban + Rural	Quantitative (Cross-sectional)	(27464; 65 +)	To examine the association between the use of alternative transportation (e.g., public transit, paratransit, getting rides, walking/wheelchair/scooter) and participation in diverse social activities among older adults aged 65 and above.
[81]	United States	Urban	Quantitative	(1221; 50 +) 64.0% were ≥ 60 years old	To explore travel behavior among older adults and identify factors that support sustainable mobility patterns, particularly in a mid-sized, auto-dependent city.
[82]	United States	Urban	Quantitative (Cross-sectional)	(455; 65–95 (73.0))	To explore places used for different social interactions that older adults engage in, particularly intergenerational interactions. To examine neighborhood environmental features linked to intergenerational interactions among older adults. To compare similarities and differences in neighborhood environmental factors associated with intergenerational interactions versus walking (for transportation or recreation).
[83]	Italy	Urban	Qualitative	(15; 60–82)	To examine how tourism impacts the everyday walking mobility and lived experiences of older residents in the historical center of Venice, a city that heavily relies on walking as a mode of transportation and tourism.
[84]	United Kingdom	Urban	Qualitative	(16; 60–87; (72.0))	To engage older adults and stakeholders to (i) identify key urban barriers and facilitators to active and healthy ageing in local urban areas of Birmingham, UK; and (ii) to build on CSS to facilitate collaboration and knowledge production to form the foundations of a network that can further purpose collective policy recommendations to promote an age-friendly society.
[85]	United Kingdom	Urban + Rural	Qualitative	(28; 65 +)	To compare determinants of outdoor trips between rural and urban-living people aged 65 and older living in England.
[86]	United Kingdom	Urban	Mixed	(173; 65 +)	To examine inequalities in perceived built environment attributes (safety, pedestrian infrastructure, and aesthetics) between high- and low-deprivation neighborhoods.
[87]	Canada	Urban + Rural	Quantitative (Cross-sectional)	(1198; 67–82; *(*73.7))	To compare the social participation of older adults living in metropolitan, urban, and rural areas, and identified associated environmental factors.
[88]	Germany	Urban + Rural	Mixed	(78; 65–92; (74.0))	To explore how community and neighborhood structures affect participation and health in older adults.

Among the 76 neighborhood attributes considered, they mainly focused on ‘Products and technology’ (32/76; 42.1%) and ‘Services, systems and policies’ (17; 22.4%), but also ‘Natural and human-made changes’ (17; 22.4%). The less frequently used attributes were ‘Support and relationships’ (8; 10.5%) and ‘Attitude’ (2; 2.6%). Attributes’ influence on mobility or social participation was mostly positive (48; 63.2%), but negative for less than the half of participants (27; 35.5%) or had no influence (1; 1.3%; Table 3). Representing 27 fewer attributes than in 2015 [11], this might be explained by 12 fewer studies retrieved in a shorter period (34 vs. 22 yrs), which provided fewer opportunities to identify attributes, and the revised search strategy. While 29 attributes remained unchanged in this update, 22 were not retrieved, 15 were added, and 32 were modified (Table 3).

Similarly to the previous review [11], mobility and social participation were both positively associated with ‘Seating’, ‘Proximity to recreational facilities’, ‘Having a car or driver’s licence’, an ‘Optimal pathway’, ‘Nature and green space’, ‘Access to amenities and services’, ‘Adequate public transportation’, ‘Neighborhood security’, and ‘Gathering places’. Moreover, mobility and social participation were both negatively associated with ‘Poor weather conditions’, ‘Inadequate public transportation’ and ‘Suboptimal pathway’. Associations also persisted with mobility, positively for ‘Aesthetics’, ‘Social cohesion’ and ‘Social support’, and negatively for ‘Inadequate lighting’, ‘Neighborhood insecurity’ and ‘Traffic’. Contrarily to the previous review [11], social participation was newly and positively associated with ‘Social cohesion’ and ‘Places of worship’ but no longer with ‘Neighborhood insecurity’. Divergent associations were found among ‘High density of population’, ‘Low neighborhood socioeconomic status’, ‘Nature and green space’, ‘Crowdedness’, ‘Adequate or Affordable public transportation’ and ‘Community based activities’.

**Table 3:** Synthesis of environmental factors linked to mobility and social participation in older adults

Environment	Mobility	Social participation
Chapter 1: Product* and technology		
e120: Products and technology for personal indoor and outdoor mobility and transportation		
Mobility assistive device	+ [60]	
e125: Products and technology for communication		
Communication technology	+ [84]	+ [52], + [80]
e140: Products and technology for culture, recreation and sport		
Community gardens	+ [55], + [70], + [81]	+ [52], + [55], + [70]
e150: Desing, construction and building products and technology of buildings for public use		
*Signage*	+ [53], + [67], + [81], + [82]	+ [82]
*Insufficient signage*	- [55], - [56]	- [55]
Parking		+ [69]
*Limited parking*	- [60], - [82]	- [72]
Seating	+ [52], + [55], + [56], + [58], 0 [62], + [70], + [73], + [82], + [67],	+ [54], + [55], + [73]
*Lack of seating*	- [55], - [60], - [64], *-* [86]	- [55]
Presence of universally accessible public spaces (railings, handrails, ramps)	+ [52], + [54]	+ [52]
Lack of universally accessible public spaces	- [55], - [83]	- [55], - [72]
Lack of washrooms facilities	- [64], - [86]	- [88]
*Picnic tables*	+ [62]	
Water fountain	+ [53], + [56]	
Weather shelter	+ [56], + [67]	+ [54]
e160: Products and technology of land development		
Aesthetics	+ [54], + [55], + [56], + [58], + [61], + [70], + [76], + [86]	+ [54], + [55], + [82]
Bridges/overpasses connecting to services	- [58], - [83]	
Crossing or crosswalks	+ [76], + [81]	
*Lack of crossing or crosswalks*		- [69], - [88]
Good user-friendliness of the walking and cycling environment including for using scooters	+ [54], + [82], + [83]	+ [54], + [68], + [82]
Poor user-friendliness of the walking and cycling environment including for using scooters	- [55], - [56], - [60]	- [55], - [69]
Presence of traffic lights	+ [76]	
Lack of traffic lights	- [86]	
Optimal pathways conditions, dimensions and coverage	+ [9], + [54], + [56], + [58], + [70], + [73], + [82]	+ [54], + [70], + [82]
Suboptimal pathways conditions, dimensions and coverage	- [9], - [52], - [55], - [58], - [60], - [62], - [86]	- [55], - [72], - [88]
Walking/cycling facilities	+ [9], + [70], + [73], + [83]	+ [64], + [73]
Rural >urban		+ [66]
Urban >rural	+ [79]	
Streets connectivity	+ [58], + [76], + [82]	+ [82]
*Unsafe stairs*	- [54], - [55], - [60]	- [55]
*Newly built neighborhood*		- [82]
*Mixed land uses and services*	+ [61], + [81]	+ [82]
Chapter 2: Natural environment and human-made changes to environment		
e210: Physical geography		
Topography physically demanding	- [52], - [60], - [64], - [70], - [85]	
*Water bodies*	+ [52]	+ [82]
e215: Population		
High density of population	- [58], + [61]	+ [57]
Low density of population	0 [82]	+ [54], + [77], + [82]
*Heterogeneity of the population*	+ [74]	+ [64]
Low neighborhood socioeconomic status	+ [79], - [86]	
Seniors’ density		+ [57], + [66]
Traffic	- [52], - [58], - [59], - [60], - [86]	- [69], - [82], - [88]
e220 Flora and fauna		
Nature and green spaces including parks	+ [55], + [56], + [58], + [60], - [62], + [66], + [70], + [71], + [73], + [81], - [82]	+ [54], + [55], + [62], - [63], + [64], + [66], + [70], + [73], - [82]
Lack of nature and green spaces including parks	- [59], - [86]	
e225: Climate		
Poor weather conditions	- [55], - [56], 0 [58], - [60], - [64], - [65], - [84], - [85]	- [54], - [55]
Good weather conditions	+ [55], + [56], + [70]	+ [55]
e240: Light		
Adequate streets and buildings lighting	+ [55], + [56], + [67], + [76]	+ [55]
Inadequate Streets and Buildings Lighting	- [56], - [58], - [60], - [67], - [70], - [86]	- [88]
e245: Time-related changes		
Nighttime	- [85]	
e250: Sound		
Absence of noise		+ [54]
e260: Air quality		
Fresh air	+ [70], + [86]	+ [70]
Chapter 3: Support and relationships		
e310: Immediate family		
Support from family		+ [68], + [85]
e320: Friends		
Support from friends		+ [68]
e325: Acquaintances, peers, colleagues, neighbours and community members		
Social cohesion	+ [53], + [56], + [58], + [60], + [70], + [78], + [85]	+ [52], + [57], + [68], + [69], + [70], + [78], + [80], + [88]
Lack of social cohesion	- [65], - [78]	- [65]
Intergenerational social engagement		+ [64]
Social support/network	+ [58], + [74], + [72], 0 [84], + [85]	+ [68], + [69], 0 [72], + [87]
Walking partner	+ [56], + [58]	+ [54]
e345: Stranger		
Crowdedness	- [58], - [83], + [86]	
Chapter 4: Attitudes		
e445: Individual attitudes of strangers		
Drivers and cyclists’ respect	+ [56]	+ [54]
Negative attitudes of people	- [56], *-* [58]	- [54], - [69]
Chapter 5: Services, systems and policies		
e510: Services, systems and policies for the production of consumer goods		
Proximity to recreational facilities	+ [52], + [54], + [55], + [70], + [83], + [85]	+ [54], + [55], + [68], + [77], + [82], + [83], + [88]
Access to amenities and services	+ [52], + [54], + [55], + [56], + [58], + [60], + [70], + [76], + [78], + [82], + [83], + [85]	+ [9], + [54], + [55], + [56], + [68], + [75], + [77], + [82], + [85], + [87]
Lack of amenities and services	- [65], - [75]	- [75], - [88]
e515 Architecture and construction services, systems and policies		
Architectural features that facilitate social contacts		+ [83]
*Lack of inclusion in deciding community infrastructure*	- [52]	
e540: Transportation services, systems and policies		
Adequate public transportation	+ [52], - [64], + [67], + [73], + [76], + [79], + [81], + [82], 0 [84], + [85]	+ [54], + [68], + [69], + [80], + [82], + [85], + [87]
Inadequate public transportation	- [60], - [75], - [65], - [79], - [67]	- [65], - [69], - [75], - [88]
*Second-hand bicycles*	+ [70]	
*Affordable public transportation*	+ [70], - [75], + [85]	+ [70]
Car or driver’s licence	+ [73], + [75], + [79], + [85]	+ [9], + [69], + [85], + [87]
e545: Civil protection services, systems and policies		
Graffiti	- [60]	
Neighborhood insecurity	- [56], - [59], - [60], + [78], - [86], - [84]	
Neighborhood security	+ [52], + [56], + [58], + [70], + [81]	+ [54], + [66], + [87]
e555 Associations and organizational services, systems and policies		
Community-based activities for older adults	+ [52], + [58], + [70], 0 [84]	+ [52], + [54], - [68], + [70], + [72]
*Places of worship*	+ [53], + [60], + [70], + [82]	+ [64], + [77], + [82], + [88]
Presence of adequate gathering places	+ [53], + [55], + [62], + [70]	+ [52], + [55], + [62], + [64], + [70], + [72], + [77], + [83], + [88]
Lack of adequate gathering places		- [9], - [72]

*Article or substance that is manufactured or refined for sale. This definition and the chapters are based on the International Classification of Functioning, Disability and Health (ICF) [31]

Attributes of the environment *in italics* were added to the scoping study of 2015 [11]

Studies considering Chap. 1 of the ICF [31], ‘Products and Technology’ mainly focused on ‘Design, construction and building products and technology of public use’ (12/32; 37.5%) and ‘Products and technology of land development’ (17; 53.1%; Table 3). More than six studies reported a positive association between mobility and social participation, and features such as the availability of seating or benches: aesthetic qualities and optimal pathway conditions, including optimal pathway dimensions and coverage. Finally, suboptimal pathway conditions, dimensions and coverage was negatively associated with both mobility and social participation (Table 3).

Among ‘Natural and human-made environment’, 3 attributes (‘Flora and Fauna’, ‘Climate’ and ‘Lights’) were equally considered (2; 11,8%) and ‘Population’ (6/17; 35,3%; Table 3). For 6 studies or more, mobility and social participation were mostly associated positively with ‘Nature and green space including parks’, while ‘Traffic’, ‘Poor weather and Conditions’ and ‘Inadequate Streets and building lighting’ were the principal attributes associated negatively with mobility and social participation.

Studies on ‘Support and Relationships’ considered mainly ‘Acquaintances, peers, colleagues, neighbours and community members’ (5/8; 62.5%; Table 3) found that ‘Social cohesion’ and ‘Social support/network’ were positively associated with both mobility and social participation for more than 6 studies. Among the few ‘Attitude’ studies, no association was made since there weren’t enough studies to establish associations.

Studies on ‘Services, systems and policies’ mainly considered ‘Transportation services, systems and policies’ (5/17; 29.4%) and ‘Associations and organizational services, systems and policies’ (4; 23.5%). Mobility and social participation were both mainly positively associated for more than 6 studies with ‘Access/proximity to recreational or exercises facilities’, ‘Presence/access of amenities and services’, ‘Adequate public transportation’, ‘Car or driver’s licence’, ‘Neighborhood security’, ‘Community-based activities for older adults’, ‘Places of worship’ and ‘Presence of adequate gathering places’. Although, they were negatively associated with ‘Inadequate public transportation’. Furthermore, mobility was negatively associated with ‘neighborhood insecurity’ (Table 3).

## Discussion

This study offered an in-depth insight into how the neighborhood environment influences mobility and social participation in older adults. Showing that mobility and social participation are highly related, often influenced by the same attributes of the environment, the present findings align with the Aging all over the place framework [89], which explicitly links neighborhood environment, mobility, and social participation, while accounting for the diversity of life trajectories. Based on an appraisal of current aging models, this framework supports the exploration of how environmental factors facilitate or constrain social engagement and mobility. Mobility is crucial and an important link between environmental conditions and social participation. Since both concepts influence each other, the association between mobility and social participation also appears to be bidirectional, which necessitate to be further studied, including for potential mediators. When mobility is restricted by the environment, participation is also indirectly limited. More specifically, mobility is highly influenced by the presence and quality of public infrastructure like seating [52], signage [53], or even sidewalks [9] and ramps [54]. These features are strongly linked with better mobility and greater social participation, as they respectively offer places to rest and make navigation easier. Safety and maintenance have also been found to be essential, as highlighted by the influence of negative conditions of the infrastructure like damaged paths [55], unsafe stairs [55], and poor lighting [56]. Such hazards not only limit capacity of some older adults to move around but also reduce confidence and willingness to go out. Population density may increase social opportunities [57] but challenges with crowdedness [58], augmentation of traffic [59], or safety concerns [60], which can reduce mobility. As shown in the previous scoping study [11], a car or driver’s licence and public transportation were both still mainly associated with mobility and social participation, supporting the importance of good and sustainable urban planning. Proximity to resources was present but did not explicitly appear in the updated results, due to its broad description being incorporated into several categories.

Nature and climate also play important roles, which might be imperative in the future as weather hazards will increase due to climate changes [90]. Green spaces [56], pleasant natural [58] and aesthetic environments [61] are generally linked positively to mobility, although some studies report mixed results [61, 78]. In general, people are more likely to go outside and engage socially when the surroundings are attractive and calming, such as with greenery [64], water features, and mild weather [65], and in absence of extreme temperatures [54] or poor weather conditions [54]. Well-designed environments, like shaded bus stops or weather shelters [53], can reduce the negative impact of weather and encourage outdoor activity and social interactions.

More articles than previously [11] also highlight the crucial role of social connections, interactions and engagements within neighborhoods, for example, with places of worship or gathering (e.g., community centres) and organized services including community-based activities. The increased influence of social cohesion may reflect studies from the COVID-19 era, when widespread isolation heightened its perceived importance, a trend that may not persist in future research. Although these organized activities motivate participation [68], groups with seniors exclusively might not be appealing for some individuals not defining themselves as older adults [68]. Connections within the community also influence the feeling of neighborhood security and sense of community, two important factors promoting walkability and urban greenway use [56], thus mobility and social participation. Conversely, perceived neighborhood insecurity negatively affects mobility [56]. Access and proximity to recreational or exercises facilities as well as amenities and services are also important for this connection, and to encourage people to go outside [54]. Sufficient and convenient local stores were crucial in 2015 [11], and still 10 years later, highlighting that their accessibility [91] and proximity [92] are key and durable determinants of mobility and social participation. Although the proportion of the population in good health remained constant [1], living in a supportive environment [1] enables people to do what is important to them, despite losses in capacity. In addition to safe and accessible public buildings and transport, and places that are easy to walk around [1], many factors can be modified to create an inclusive community where older adults live safely, enjoy good health and stay involved. Since greater social participation and fewer environmental barriers best predict quality of life [93], a public-health response to aging should consider individual and environmental approaches that reduce the losses associated with older age but reinforce recovery, adaptation and psychosocial growth [1]. Age-friendlier environments are not only physically accessible spaces but emancipating, welcoming and inclusive communities.

Through a clearly defined extensive and systematic search strategy in several multidisciplinary databases and in-deep analysis guided by a worldwide renowned classification, this review updated a previous scoping study [11] by identifying several recent empirical studies (38), proving rigorously new results supported. Based on 16 qualitative, 12 quantitative and 10 mixed studies, these results complete and expand the understanding of how the neighborhood environment influence mobility and social participation. Despite the relative heterogeneity of the included studies, and that all older adult’s experiences are different, certain groups such as people aged 80 or over [69], those having low income [70] or disabilities secondary to physical [71] or mental health [59] diseases, those living in rural areas [72], or belonging to an ethnic [73], linguistic, sexual or gender [74] minority group, may face other challenges and experience additional barriers to mobility and social participation [94], that need to be further studied.

Among limitations, the selected studies were composed of several qualitative and only one clinical randomized trial, preventing causality assessment, and presenting essential preliminary steps but a low quality and level of evidence requiring at least 6 studies supporting one specific item to be considered a meaningful result. Furthermore, such heterogeneity may limit studies’ comparability. Despite a careful and collaborative review process involving a research team of 7 members, relevant studies on the neighborhood environment, mobility and social participation might have been missed, especially if focusing on specific topics or populations. Furthermore, time constraints restrict the possibility of expanding the scope of this study and including knowledge-users, as recently recommended in the field [95].

Future research should prioritize longitudinal designs, to gain a better understanding of causality between neighborhood environments, mobility and social participation. Additionally, intervention studies are required to assess the effectiveness of targeted changes in the built or social environment. With knowledge-users, such as community members and clinicians, co-construction of more inclusive environment that fosters mobility and social participation is also recommended to enhance relevance and applicability of findings. Finally, given the underrepresentation of some groups in literature, future studies should ensure inclusive sampling that reflects diverse populations, including people aged 80 or over, those having low income or disabilities secondary to physical or mental health diseases, those living in rural areas, or belonging to an ethnic, linguistic, sexual or gender minority group.

## Conclusion

This comprehensive synthesis of empirical studies aimed to update existing knowledge on how neighborhood environment influences mobility and social participation among older adults. The results support the development of innovative interventions, clear guidelines, and planning greater practices addressing notably ‘Aesthetics’, ‘Nature and green space’, ‘Social cohesion’, ‘Presence/access of amenities and services’, ‘Adequate public transportation’, and ‘Neighborhood security’. Since fewer studies targeted ‘Attitudes’ among older adults, further research is needed to better understand how mobility and social participation are associated with this attribute. As neighborhood dynamics vary greatly and influence older adults’ daily lives in distinct ways, it is important to consider the diversity of living contexts when analyzing study’s data and planning future research. Considering that most of the studies concerned urban environments, exploring a wider range of settings (e.g., rural and suburban) for a future study may lead to valuable comparisons. Future studies should also focus on widely appreciated categories such as parks, sidewalk condition or gathering spaces, by examining more precisely how and which dimensions are linked mobility and social participation or were influenced by the COVID-19 pandemic. Continued investigation into neighborhoods, mobility and participation can provide important insights for the development of age-friendly environments and promotion of community engagement.

## Data Availability

Not applicable.

## Appendix 1

Definitions of environmental factors according to the International Classification of Functioning, Disability and Health (ICF) [31].

### Chapter 1: products and technology

This chapter is about the natural or human-made products or systems of products, equipment and technology in an individual’s immediate environment that are gathered, created, produced or manufactured. The ISO 9999 classification of technical aids defines these as ‘any product, instrument, equipment or technical system used by a disabled person, specially produced or generally available, preventing, compensating, monitoring, relieving or neutralizing’ disability. It is recognized that any product or technology can be assistive. (See ISO 9999: Technical aids for disabled persons, Classification (second version); ISO/TC 173/SC 2; ISO/DIS 9999 (rev.).) For the purposes of this classification of environmental factors, however, assistive products and technology are defined more narrowly as any product, instrument, equipment or technology adapted or specially designed for improving the functioning of a disabled person.

### Chapter 2: natural environment and human-made changes to environment

This chapter is about animate and inanimate elements of the natural or physical environment, and components of that environment that have been modified by people, as well as characteristics of human populations within that environment.

### Chapter 3: support and relationships

This chapter is about people or animals that provide practical physical or emotional support, nurturing, protection, assistance and relationships to other persons, in their home, place of work, school or at play or in other aspects of their daily activities. The chapter does not encompass the attitudes of the person or people that are providing the support. The environmental factor being described is not the person or animal, but the amount of physical and emotional support the person or animal provides.

### Chapter 4: attitudes

This chapter is about the attitudes that are the observable consequences of customs, practices, ideologies, values, norms, factual beliefs and religious beliefs. These attitudes influence individual behaviour and social life at all levels, from interpersonal relationships and community associations to political, economic and legal structures; for example, individual or societal attitudes about a person’s trustworthiness and value as a human being that may motivate positive, honorific practices or negative and discriminatory practices (e.g. stigmatizing, stereotyping and marginalizing or neglect of the person). The attitudes classified are those of people external to the person whose situation is being described. They are not those of the person themselves. The individual attitudes are categorized according to the kinds of relationships listed in Environmental Factors Chap. 3. Values and beliefs are not coded separately from the attitudes as they are assumed to be the driving forces behind the attitudes.

### Chapter 5: services, systems and policies

This chapter is about:

1. Services that provide benefits, structured programmes and operations, in various sectors of society, designed to meet the needs of individuals. (Included in services are the people who provide them.) Services may be public, private or voluntary, and may be established at a local, community, regional, state, provincial, national or international level by individuals, associations, organizations, agencies or governments. The goods provided by these services may be general or adapted and specially designed.
2. Systems that are administrative control and organizational mechanisms, and are established by governments at the local, regional, national, and international levels, or by other recognized authorities. These systems are designed to organize, control and monitor services that provide benefits, structured programmes and operations in various sectors of society.
3. Policies constituted by rules, regulations, conventions and standards established by governments at the local, regional, national, and international levels, or by other recognized authorities. Policies govern and regulate the systems that organize, control and monitor services, structured programmes and operations in various sectors of society.

Based on the International Classification of Functioning, Disability and Health (ICF) [31] this the list of attributes of each chapters:

### Chapter 1: product and technology

- e110: Products or substances for personal consumption.
- e115: Products and technology for personal use in daily living.
- e130: Products and technology for education.
- e135: Products and technology for employment.
- e145: Products and technology for the practice of religion and spirituality.

### Chapter 2: natural environment and human-made changes to environment

- e230: Natural events.
- e235: Human-caused events.
- e255: Vibration.

### Chapter 3: support and relationships

- e315: Extended family.
- e330: People in positions of authority.
- e335: People in subordinate positions.
- e340: Personal care providers and personal assistants.
- e355: Health professionals.
- e360: Other professionals.

### Chapter 4: attitudes

- e410: Individual attitudes of immediate family members.
- e415: Individual attitudes of extended family members.
- e420: Individual attitudes of friends.
- e425: Individual attitudes of acquaintances, peers, colleagues, neighbours and community members.
- e430: Individual attitudes of people in positions of authority.
- e435: Individual attitudes of people in subordinate positions.
- e440: Individual attitudes of personal care providers and personal assistants.
- e450: Individual attitudes of health professionals.
- e455: Individual attitudes of health-related professionals.

### Chapter 5: services, systems and policies

- e510: Services, systems and policies for the production of consumer goods.
- e520: Open space planning services, systems and policies.
- e530: Utilities services, systems and policies.
- e535: Communication services, systems and policies.
- e550: Legal services, systems and policies.
- e565: Economic services, systems and policies.
- e570: Social security services, systems and policies.
- e575: General social support services, systems and policies.
- e585: Education and training services, systems and policies.
- e590: Labour and employment services, systems and policies.
- e595: Political services, systems and policies.
